# Compression médullaire d'origine métastatique

**DOI:** 10.11604/pamj.2014.19.209.3695

**Published:** 2014-10-27

**Authors:** Touria Bouhafa, Abderrahman Elmazghi, Ouafae Masbah, Khalid Hassouni

**Affiliations:** 1 Service de Radiothérapie, CHU Hassan II de FES, Faculté de Médecine et de Pharmacie de Fès, Fès, Maroc

**Keywords:** Compression médullaire, métastase, traitement corticothérapie, chirurgie, radiothérapie, Spinal cord compression, metastases, corticotherapy, surgery, radiotherapy

## Abstract

La compression médullaire d'origine métastatique est une complication neurologique fréquente du cancer. C'est une urgence diagnostique et thérapeutique qui nécessite une prise en charge rapide et efficace. L'imagerie par résonnance magnétique (IRM) constitue l'examen de choix pour l'exploration de l'ensemble de la moelle. La prise en charge thérapeutique doit être multidisciplinaire incluant la corticothérapie, la radiothérapie et la chirurgie.

## Introduction

La compression médullaire d'origine métastatique constitue une urgence oncologique. En l'absence de prise en charge thérapeutique, cette affection risque d'entraîner, à court ou à moyen terme, une paraparésie et éventuellement une paraplégie, des déficits sensitifs et une atteinte sphinctérienne [1, 2]. Dans 75% des cas, la compression médullaire est causée par une masse tumorale osseuse envahissant le canal spinal, situation fréquente car entre 40 et 70% des patients présentant une tumeur solide développeront des métastases osseuses à des stades avancés [1, 3]. Le traitement de la compression médullaire se base sur la corticothérapie, la radiothérapie et la chirurgie. À travers une revue de la littérature ne discutons les données cliniques, radiologiques et thérapeutiques de cette pathologie fréquemment rencontré en pratique clinique et dont sa prise en charge constitue un défi pour les cliniciens.

## Méthodes

Une recherche sur PubMed et sur ScienceDirect en utilisant les mots clés " prise en charge de la compression médullaire non traumatique", a été réalisée, ce qui a permis d′obtenir une vision globale sur cette pathologique, sa fréquence, les moyens diagnostiques ainsi que les différentes armes thérapeutiques, les articles les plus pertinents sont retenus.

## Etat actuel des connaissances

### Épidémiologie

Les métastases issues de cancers du sein, de la prostate et du poumon sont responsables de la majorité des cas de compression médullaire (chaque type histologique cause environ de 15 à 20% des cas) [1, 4, 5]. La compression dans cette situation, est causée par l'envahissement de l'espace péridural par une lésion métastatique localisée initialement au niveau d'une vertèbre [6, 7]. D'autres types de cancer comme les cancers du rein, les lymphomes ou les myélomes peuvent aussi provoquer cette complication (de 5 à 10% des cas), généralement attribuable à la progression d'une lésion néoplasique pararachidienne qui a infiltré les trous de conjugaison avant d'envahir l'espace péridural [5–7].

La compression de la moelle épinière survient le plus fréquemment au niveau thoracique (de 60% à 80%), chez 15% à 30% des patients, l'atteinte se situera au niveau lombosacré et dans moins de 10% des cas, au niveau cervical [1, 5, 7]. La facilité avec laquelle les métastases se développent au niveau de la colonne vertébrale s'expliquerait par le fait qu'il s'agit d'une région osseuse très vascularisée et riche en facteurs de croissance [1, 5, 8]. Des dommages médullaires tels que la congestion vasculaire, les hémorragies, l'oedème de la substance blanche et les dommages neuronaux sont fréquemment observés au site de la compression médullaire [1, 4, 5, 9].

### Les manifestations cliniques de la compression médullaire

#### Douleur

Près de 90% des patients ressentiront une douleur au moment du diagnostic. Toutefois, le fait que cette douleur peut s'exprimer de différentes façons complique le diagnostic. La douleur peut être localisée au niveau rachidien ou pararachidien et être associée ou non à une douleur radiculaire. Occasionnellement, les patients signalent une douleur qui ne respecte pas les dermatomes habituels (referred pain) [1, 2, 5, 7]. Une douleur mécanique peut aussi survenir lorsque le patient présente un écrasement vertébral ou une instabilité de la colonne vertébrale [1]. Bien que la douleur ne soit pas considérée, en soi, comme un facteur prédictif de compression de la moelle épinière [2, 6], plusieurs cliniciens estiment que l'apparition d'une nouvelle douleur ou la modification de l'intensité ou des caractéristiques d'une douleur existante doit évoquer la possibilité d'une compression médullaire [6, 10]. De plus, différentes études ont montré que la douleur précède souvent de plusieurs semaines, voire de quelques mois, le début de l'atteinte neurologique [5–7, 10].

### Atteinte motrice, atteinte sensitive et troubles sphinctériens

Une des conséquences les plus dramatiques de la compression médullaire est le développement d'un déficit neurologique moteur ou sensitif. Lors du diagnostic, entre 35 et 85% des patients présentent ce symptôme [4–6, 11]. Dans les faits, l’état neurologique moteur au moment du traitement est le facteur prédictif le plus important de la fonction motrice après le traitement [2, 5, 6], lorsqu'on compile les données de plusieurs études, on constate que près de 94% des patients qui sont encore capables de marcher au moment du diagnostic conserveront leur capacité motrice après la radiothérapie alors que seulement 13% des personnes paraplégiques avant la radiothérapie marcheront de nouveau après le traitement [2, 6]. Ces résultats mettent en évidence l'importance de poser non seulement un diagnostic précoce, mais surtout de soupçonner rapidement la présence d'une atteinte motrice.

Malheureusement, un délai entre la présentation des symptômes et le diagnostic est fréquent, les symptômes ressentis étant souvent considérés comme non spécifiques [12, 13]. Enfin, la rapidité d'installation du déficit moteur semble également être un facteur prédictif de l’état fonctionnel du patient après le traitement. Ainsi, les personnes chez qui le déficit moteur s'installe « lentement» (sur une période de plus de 14 jours) semblent avoir plus de chances de noter une amélioration de leur état fonctionnel [2, 6]. D'autres symptômes tels que la dysfonction des sphincters, trouvé chez près de la moitié des patients, ou l'ataxie peuvent être aussi observés [1, 5–7].

### Diagnostic radiologique de la compression médullaire

La présence des signes clinique en faveur de la compression médullaire constitue une urgence oncologique et nécessite une prise en charge immédiate, ainsi l'imagerie par résonnance magnétique (IRM) constitue la méthode de choix pour la détection de la compression médullaire (sensibilité: 93%, spécificité: 97%, efficacité: 95%) [1, 11, 14, 15]. Cet examen est non invasive permet de détecter très précocement un syndrome subclinique, c'est-à-dire un début de compression du sac thécal sans symptômes cliniques francs. Il permet également de préciser les niveaux segmentaires atteints et la nature de la compression et d'exclure d'autres diagnostics potentiels. L'ensemble du rachis et la moelle sont explorés en séquences sagittales, axiales et parfois coronales pondérées en T1 et T2, L'IRM permettra de faire une cartographie du processus lésionnel en déterminant: le siège de la lésion en hauteur (cervical, thoracique, lombaire; la localisation dans un espace rachidien (extradural, intradural extramédullaire ou intramédullaire); le nombre, l’étendue et les dimensions de la lésion; les rapports avec les structures avoisinantes; les caractères sémiologiques de la lésion; le retentissement sur la moelle [16].

L'examen par tomodensitométrie est moins sensible et moins informatif que la résonance magnétique, mais l'arrivée des appareils multibarrettes a permis d'en accroître la précision. Cet examen est efficace pour évaluer l’étendue de l'atteinte métastatique osseuse et permet de repérer des foyers d'envahissement, surtout s'ils sont à point de départ osseux. Par contre, cet examen ne permet pas de bien visualiser la morphologie de la moelle épinière [6]. Une radiographie simple est d'une utilité relativement limitée, elle peut nous révéler une lyse osseuse ou un tassement vertébral, ce qui viendrait soutenir notre hypothèse diagnostique. Par contre, un résultat négatif ne permet pas d'exclure la présence d'un envahissement osseux, ni d'une compression. L'IRM est l'examen de choix en cas de suspicion d'une compression médullaire et les autre moyens d'imagerie ne seront utiliser que si l'IRM est contre-indiquée ou non disponible selon les milieux hospitaliers [5].

### Modalités thérapeutiques

Le choix de l'approche thérapeutique doit tenir compte de plusieurs éléments, tels que le statut ambulatoire du patient, l'histopathologie de la tumeur primaire, la rapidité du développement d'un déficit neurologique, l'activité systémique de la tumeur primaire (sous contrôle ou non), l’âge, le statut de performance, le nombre de niveaux de compression médullaire lors du diagnostic et le délai entre le diagnostic de la tumeur primaire et la compression médullaire [1, 4, 5, 13, 14, 17–19]. Dans ce contexte le but du traitement est d'améliorer la fonction neurologique, surtout l'autonomie à la marche, de diminuer la douleur et de préserver ou d'améliorer la qualité de vie [1]. Trois armes thérapeutiques sont disponibles pour le traitement de la compression médullaire soient la corticothérapie, la radiothérapie et la chirurgie décompressive et de stabilisation.

### La corticothérapie

L'utilisation de la corticothérapie dans le cadre du traitement de première intention de la compression médullaire est répandue. La dexaméthasone est le corticostéroïde le plus souvent utilisé. Cette thérapie agit au niveau de la réduction de l'oedème, de l'inhibition de la réponse inflammatoire, de la stabilisation des membranes vasculaires et conséquemment, elle agit au niveau de la réduction de la douleur, sur le délai avant l'apparition de déficit neurologique et sur l'amélioration de symptômes déjà présents [1, 5]. Bien que plusieurs schémas posologiques aient été suggérés, il n'existe pas, en ce moment, assez de données pour guider notre choix, ni la posologie optimale, ni la durée d'administration idéale ne sont connues [2, 6]. En pratique, plusieurs cliniciens administrent une dose initiale de 10 mg de dexaméthasone, suivie de 4 mg toutes les six heures pendant quelques jours, puis de doses décroissantes. En présence de signes cliniques francs de myélopathie ou d'une évolution rapide de l'atteinte neurologique, certains utilisent une dose initiale plus élevée, allant jusqu’à 100 mg, comme dose de charge, suivie de 24 mg toutes les six heures, puis de doses décroissantes [5, 10].

Pour les patients ne présentant qu'un syndrome subclinique, il n'y a pas de données solides qui nous permettent de déterminer si l'administration prophylactique de corticostéroïdes est bénéfique ou non [2, 5]. Il est important de s'assurer de bien soulager la douleur généralement associée à ce syndrome. On trouve souvent une douleur de type mixte avec une composante nociceptive (à la suite de l'envahissement osseux) et une composante neuropathique (à la suite de l'atteinte nerveuse) [6, 7, 13].

### Radiothérapie

La radiothérapie est l'arme thérapeutique majeure dans le traitement de la compression médullaire. Toutefois, le traitement optimal n'est pas bien défini [1, 2, 11, 14]. Actuellement, particulièrement en Amérique du Nord, une dose totale de 30 Gy en 10 fractions constitue un des régimes les plus utilisés [1, 4, 9]. En Europe, des régimes plus courts (une dose de 8 Gy ou 20 Gy en 5 fractions) sont aussi fréquemment utilisés [4]. Rades et al. ont publié en 2011 les résultats d'une étude de phase II multicentrique internationale comparant l'efficacité d'un régime court de radiothérapie à un régime long pour le contrôle local de la compression médullaire (données probantes de niveau III) [20]. Au total 265 patients présentant un déficit moteur des membres inférieurs résultant d'une compression médullaire, confirmée par IRM, les patients ont reçu: un régime court (8 Gy X 1 ou 4 Gy X 5) (n = 131); un régime long (3 Gy X 10 ou 2,5 Gy X 15 ou 2 Gy X 20) (n = 134).

Tous les patients ont reçu de la dexaméthasone dès le premier jour de radiothérapie. Les caractéristiques des patients étaient similaires dans les deux groupes. Le suivi médian a été de 13 mois. Le contrôle local à 12 mois a été significativement supérieur suivant un régime long de radiothérapie comparativement à un régime court (81% contre 61%; p = 0,005). Pour la fonction motrice et la survie, aucune différence significative n'a été observée entre les différents types de régime court (p = 0,61) ou long (p = 0,37). Cependant l'analyse multivariée a permis de déterminer que la survie était significativement associée au statut de performance de l'ECOG, au nombre de vertèbres impliquées, à la présence de métastases viscérales, au statut ambulatoire pré-radiothérapie et à l'administration de bisphosphonates après la radiothérapie. Les auteurs de cet article proposent la réalisation du protocole long de la radiothérapie pour le groupe de malade avec un pronostic de survie relativement favorable. Une autre étude phase II intéressante publiée en 2004 par Rades et al. Qui a évalué l'efficacité de deux régimes longs de radiothérapie pour le traitement de la compression médullaire (données probantes de niveau III) [21]. Un total de 214 patients ont été inclus. Selon la disponibilité de l'appareillage, les patients ont reçu une dose totale de 30 Gy donnée en 10 fractions de 3 Gy chacune (n = 110) ou une dose totale de 40 Gy donnée en 20 fractions de 2 Gy chacune (n = 104). Le suivi médian a été de 6 mois. Aucune différence significative sur le statut ambulatoire ni sur les effets indésirables liés au traitement n'a été observé entre les deux régimes. Une analyse multivariée a démontré qu'une progression lente du déficit moteur, une histologie tumorale favorable et le fait d’être ambulant avant le traitement étaient significativement associés à une meilleure efficacité de la radiothérapie (p < 0,035).

De façon générale, les champs d'irradiation englobent le site de la compression avec une à deux vertèbres au-dessus et en dessous de la lésion à traiter [22]. Dans les cas oùun nombre limité de vertèbres serait impliqué et que le pronostic de survie du patient serait relativement favorable, une dose de radiothérapie peut être administrée directement aux vertèbres concernées en laissant une marge de sécurité de 1 à 2 cm [22]. Bien qu'il existe de grandes variations dans la pratique clinique actuelle concernant la modalité de l'irradiation, une évaluation précise du tableau clinique (déficit neurologique déjà installé ou juste une présentation subclinique de la compression médullaire), l’état général du malade, la radiosensibilité de la tumeur primaire, l'espérance de vie, le nombre et l'emplacement des lésions compressives, le niveau de risque chirurgical, est primordial pour décider la modalité de la radiothérapie a proposé au patient [2, 12, 23].

Actuellement, de nouvelles techniques commencent à être de plus en plus utilisées telles que la radiochiurgie stéréotaxique et la radiothérapie avec modulation d'intensité (IMRT), permettant une haute précision avec des doses plus élevées directement sur la cible, tout en épargnant la moelle normale et les tissus paraspinaux, avec des résultats très prometteurs [24, 25]. Cependant, peu d’études ont jusqu'ici directement comparé l'efficacité et la toxicité de la radiochirurgie au autres types de radiothérapie.

### Place de la chirurgie décompressive

Le rôle de la chirurgie décompressive demeure mal défini. Les avantages de cette technique incluent la cytoréduction maximale de la métastase, le soulagement immédiat de la compression médullaire, le retrait de fragments osseux si nécessaire ainsi que la stabilisation de la colonne. Trois études phase II ont évalué l'impact de la chirurgie sur la qualité de vie (Wu et al. [26], Falicov et al. [27], Wai et al. [28]) elles ont montré une amélioration significative de la qualité de vie et de la douleur.

### Association chirurgie et RTH

Klimo et al. ont publié en 2005 une méta-analyse comparant l'efficacité d'une chirurgie combinée à une radiothérapie à celle d'une radiothérapie seule pour le traitement de la compression médullaire, l'association des deux armes therapeutique permettait de préserver la marche après traitement (1,3 fois plus que la RTH seule) et de regagner une fonction motrice normale (deux fois plus) [25, 29]. Cependant, ces résultats dépendaient essentiellement de l’état général des patients, de l’étendue des lésions, de la tumeur primitive et de la fonction motrice avant traitement. Patchell et al. Ont publié les résultats d'un essai prospectif de phase III randomisé incluant 101 patients comparant la place de la chirurgie associée à la radiothérapie versus radiothérapie seule [30]. L'objectif primaire dans cet essai était l’évaluation de la fonction motrice (marche) après le traitement. Tous les patients de l’étude ont reçu une dose de charge de 100 mg de dexaméthasone puis 24 mg toutes les six heures avec réduction de la dose jusqu’à la fin du traitement, le schéma de la radiothérapie utilisé est de 30 Gy en dix fractions.

La chirurgie suivie de RTH avait permis à 84% des patients de préserver la capacité de marcher versus 57% dans le bras RTH seule, et cela de manière significative (p = 0,003). Sur 32 patients paraplégiques, 62% avaient récupéré la marche après traitement combiné contre 19% seulement après RTH seule (p = 0,012) [30]. Patchell et al. avaient, certes, pu démontrer l'intérêt d'associer une chirurgie à la RTH, cependant pour des patients très sélectionnés. Mais aucun essai prospectif randomisé n'a jusqu'ici comparé la chirurgie seule à la chirurgie suivie d'une RTH [25].

### Chimiothérapie, hormonothérapie et les bisphosphonates

Les bisphosphonates tels que l'acide zolédronique et le pamidronate ont prouvé leurs apports dans l'amélioration de la qualité de vie en agissant sur la douleur. Ils diminuent aussi la fréquence des événements osseux. La place de la chimiothérapie et l'hormonothérapie dans le traitement de la compression médullaire est difficile a évalué vu l'absence des essais contrôlés randomisés, néanmoins ces options peuvent être envisagées dans des cas précis; les tumeurs chimiosensibles (maladie de Hodgkin et les lymphomes non hodgkiniens, les tumeurs germinales) ou encore hormonosensible (cancer du sein avec récepteurs hormonaux fortement positives, cancer de la prostate); en plus des autres approches thérapeutiques décrites précédemment [25, 31–33].

## Conclusion

La compression médullaire d'origine métastatique est une situation fréquemment rencontré en oncologie, c'est une très grande urgence diagnostique et thérapeutique, tout retard peut engendrer le pronostic fonctionnel, et aggraver la qualité de vie des malades, sa prise en charge se base sur une attitude multidisciplinaire incluant le radiologue, le chirurgien, l'oncologue et le radiothérapeute.
